# Plasma IL-5 but Not CXCL13 Correlates With Neutralization Breadth in HIV-Infected Children

**DOI:** 10.3389/fimmu.2019.01497

**Published:** 2019-07-02

**Authors:** Julia Roider, J. Zachary Porterfield, Paul Ogongo, Maximilian Muenchhoff, Emily Adland, Andreas Groll, Lynn Morris, Penny L. Moore, Thumbi Ndung'u, Henrik Kløverpris, Philip J. R. Goulder, Alasdair Leslie

**Affiliations:** ^1^Africa Health Research Institute (AHRI), Nelson R. Mandela School of Medicine, University of KwaZulu-Natal, Durban, South Africa; ^2^Department of Paediatrics, University of Oxford, Oxford, United Kingdom; ^3^HIV Pathogenesis Programme, Doris Duke Medical Research Institute, Nelson R. Mandela School of Medicine, University of KwaZulu-Natal, Durban, South Africa; ^4^Department of Infectious Diseases, Ludwig-Maximilians-University, Munich, Germany; ^5^German Center for Infection Research (DZIF), Munich, Germany; ^6^School of Public Health, Yale University, New Haven, CT, United States; ^7^School of Laboratory Medicine and Medical Sciences, University of KwaZulu-Natal, Durban, South Africa; ^8^Institute of Primate Research, National Museums of Kenya, Nairobi, Kenya; ^9^Faculty of Medicine, Virology, National Reference Center for Retroviruses, Max von Pettenkofer Institute, LMU München, Munich, Germany; ^10^Faculty of Statistics, TU Dortmund University, Dortmund, Germany; ^11^Centre for HIV and STIs, National Institute for Communicable Diseases of the National Health Laboratory Service, Johannesburg, South Africa; ^12^Faculty of Health Sciences, University of the Witwatersrand, Johannesburg, South Africa; ^13^Center for the AIDS Programme of Research in South Africa (CAPRISA), Durban, South Africa; ^14^Department of Infection and Immunity, University College London, London, United Kingdom; ^15^Virology and Immunology, Max Planck Institute for Infection Biology, Berlin, Germany; ^16^The Ragon Institute of Massachusetts General Hospital, Massachusetts Institute of Technology and Harvard University, Boston, MA, United States; ^17^Department of Immunology and Microbiology, University of Copenhagen, Copenhagen, Denmark

**Keywords:** CXCL13, IL-5, Activin A, broadly neutralizing antibodies (bnAbs), pediatric HIV, plasma markers, HIV neutralization breadth, T-follicular helper cells (Tfh)

## Abstract

Children may be the optimal target for HIV vaccine development as they generate substantially more frequent and more potent broadly HIV neutralizing antibodies (bnAbs) than adults. Development of a biomarker that correlates with neutralization breadth in this group could function as a powerful tool to facilitate the development of an HIV vaccine. Previously, we observed that this preferential ability in HIV-infected children over adults to generate bnAbs is associated with an enrichment of circulating follicular helper T-cells (T_FH_) with an effector phenotype, and the presence of IL-21 secreting HIV-specific T_FH_ within lymphoid tissue germinal centers (GC). In adults, bnAbs development has been linked with high plasma levels of CXCL13, a chemoattractant for CXCR5-expressing T_FH_ cells to the lymph node GC. We sought to test this relationship in HIV-infected children, but found no association between neutralization breadth and plasma levels of CXCL13, or with the Th2 cytokines IL-4 and IL-13, or the T_FH_ associated factor Activin A. However, we did find an unexpected association between plasma IL-5 levels and bnAb development in these children. Importantly, although CXCL13 correlated with total circulating T_FH_ cells, it was not associated with effector T_FH_. Additionally, raised CXCL13 expression was associated with a lower CD4 percentage, higher viral load and a loss of immune function, implying it is associated with progressive disease rather than HIV-specific GC activity in these subjects. Taken together, our data suggests that IL-5 should be evaluated further as a candidate plasma biomarker for HIV neutralization breadth and for monitoring vaccine responses in the pediatric age group.

## Introduction

Recent HIV vaccine development strategies have highlighted the unique potential contribution that can be made by the study of the immune responses to HIV infection in children (1). In response to the same gp120 vaccine, infants produced higher magnitude anti-V1V2 antibodies than adults (2). Furthermore, there is growing evidence that children are better at eliciting HIV-specific antibody responses than adults and develop broad and potent neutralizing antibodies as early as 2 years of life (3, 4). Although the mechanistic details are not fully established, the immune system in early life is specifically adapted, via the support and regulation of T_FH_ activity, to high frequency, high affinity antibody production (5, 6).

Germinal centers (GC) of secondary lymphoid organs are the primary site where a humoral immune response develops, but are challenging to directly monitor in vaccine trials due to a lack of routine access to lymphoid tissue. Recent attempts to use fine needle aspirates show promise (7), but this invasive technique is unlikely to be adopted for children. As an alternative various studies of plasma markers of germinal center activity have been conducted in adults to seek for correlates to GC activity (8–10).

T-follicular helper cells (T_FH_) are key regulators of the antibody immunity, as they are critical for the formation of GCs and for the development of high-affinity antibodies and memory B cells. They restrict access to GCs for low-affinity B cells, promote the survival of high-affinity B cells and trigger B cell receptor (BCR) signaling. Crucially, signaling from T_FH_ cells drives the extensive somatic hypermutation necessary for the generation of broadly neutralizing antibodies to HIV (11–13). Although T_FH_ cells mainly function in secondary lymphoid tissue, they are also present in circulation and have been associated with HIV neutralization breadth in adult (14) and pediatric (6) infection. However, it remains unclear how the frequency and phenotype of these cells in circulation reflects their activity within the GC. Moreover, we have previously reported the presence of high frequency IL-21 producing HIV-specific GC T_FH_ in lymphoid tissue of HIV-infected children that are not readily detected in circulation (6). An alternative approach is to study plasma markers that might correlate with T_FH_ activity within the GC. The CXCL13–CXCR5 chemokine axis, for example, plays a central role in organizing both B-cell follicles and GCs, as CXCR5 expressing T_FH_ cells use CXCL13 to migrate into the GCs of secondary lymphoid tissue (15). In adults, it has been reported that plasma CXCL13 levels during early HIV infection can predict the generation of broadly neutralizing antibodies in chronic disease (8, 10). Additionally, elevated plasma levels of CXCL13 have been observed in HIV-infected individuals with high neutralization breadth (9). Additionally, a positive correlation between circulating T_FH_ and plasma CXCL13 levels has been observed in HIV-infected children (16). However, whether CXCL13 correlates with GC activity or the development of bnAbs against HIV in children is unknown. Using a previously described cohort of HIV-infected children, in whom the frequent development of bnAbs was associated with the T_FH_ response (6), we therefore tested the potential role of CXCL13 as a biomarker of increased T_FH_ activity and neutralization breadth. In addition, we measured other candidate plasma markers of T_FH_ activity: Activin A has been identified as a powerful regulator of the differentiation of T_FH_ cells, which is antagonized by IL-2 (17); and the Th2 cytokines IL-13, IL-4, and IL-5. The latter two have been described as important stimulators of the GC reaction (18), and impaired IL-5 production by T-cells has been previously described as a prognostic marker of disease progression in HIV-infected children (19).

## Materials and Methods

### Study Participants

Plasma and peripheral blood mononuclear cells (PBMC) of 45 vertically HIV-1 C clade-infected ART-naïve children with matched neutralization data (4) and of pediatric uninfected controls (*n* = 7) were studied. All children were recruited from South African clinics at Kimberley Hospital (Kimberley, South Africa) and Ithembalabantu Clinic (Durban, South Africa). Uninfected controls were from the same ethnical background, being mostly siblings of infected study participants. Notably, the uninfected controls available for this study were significantly older than the HIV-infected children studied (13-16.5 vs. 5.7-10.8 years old). The clinical characteristics of the study cohort are shown in Table 1. “High” neutralizers were defined by neutralization of ≥81% of tested viruses (≥75% percentile; *n* = 13) and “low” neutralizers by neutralization of ≤44% of tested viruses (≤25% percentile; *n* = 13). Viral load measurements were performed as described previously (4). Informed consent was obtained from all adult study participants or from the caregivers of pediatric participants where appropriate. Additionally, assent to participate in the study was given directly by children from the age of 6 and above. Studies were approved by the University of the Free State Ethics Committee, Bloemfontein; Biomedical Research Ethics Committee, University of KwaZulu-Natal, Durban; and Research Ethics Committee, University of Oxford.

**Table 1:** Clinical characteristics of study cohort.

**Group**	***n***	**Age median (IQR)**	**CD4/mm^**3**^ median (IQR)**	**VL cp/ml median (IQR)**	**Neutralization breadth (IQR)**
Pediatric infected	45	7.6 (6.1–9.8)	790 (309–1067)	42,000 (14,000–132,834)	63 (44–81)
high neutralizers (high)	13	9.6 (6.6–10.8)	662 (315–1021)	62,000 (17,184–104,712)	88 (81–97)
low neutralizers (low)	13	7.0 (5.7–10.2)	1039 (806–1101)	37,207 (7080–194,243)	38 (22–44)
Pediatric uninfected	7	15.0 (13.0–16.5)	N/A	N/A	N/A
					N/A not applicable

### Sample Processing—PBMC and Plasma

Plasma was separated by centrifugation and crypopreserved at −80°C. PBMCs were isolated by Ficoll density gradient centrifugation and stored in liquid nitrogen until use.

### Virus Neutralization Assays

The ability of plasma from infected children to neutralize HIV was measured against a panel of 16 tier 2 and tier 3 clade A, B, and C viruses. Neutralization was determined by a reduction in luciferase gene expression after a single round of infection of JC53bl-13 cells, also known as TZM-bl cells (National Institutes of Health AIDS Research and Reference Reagent Program), with Env-pseudotyped viruses as previously described (4). Titer was calculated as the reciprocal plasma/serum dilution causing a 50% reduction of relative light units [median infective dose (ID50)].

### Flow Cytometry and ICS Assays

PBMCs obtained at the same time point as plasma were stained with fluorescent monoclonal antibodies against markers associated with T_FH_ cells as previously described (6). For a subset of this cohort (*n* = 18), intracellular cytokine staining assays for IL-4 (BD, FITC, 554484), IL-5 (BD, APC, 554397), IL-13 (BD, V450, 561158), TNF-α (BD, AF700, 557996), and INF-γ (BD, PE-Cy7, 557643) were performed. Briefly, cells were stimulated with PMA/Ionomycin (at a final concentration of 4 ×10^−5^M) in the presence of anti-CD28 and anti-CD49 (1 mg/ml), Brefeldin A and Monensin (5 mg/ml) (BD biosciences) for 5 h, followed by surface staining and intracellular staining. Rainbow beads (BD biosciences) were run with every experiment and compensation was adjusted to ensure longitudinal comparability of experiments. Flow cytometry acquisition was performed on a BD LSRFortessa within 5 h of staining and analyzed using FlowJo version 9.9.5.

### Plasma Assays

Plasma markers and neutralization breadth were determined in matching time points in vertically infected children for all available samples. *Ex-vivo* plasma levels of IL-5 were quantified using a commercially available Luminex kit for human cytokine/chemokine (Milliplex). Plasma samples were tested in duplicate per the manufacturer's recommendations. Plasma levels of CXCL13 were quantified using a commercially available enzyme-linked immunosorbent assay kit (R&D Systems) in duplicate. Plasma levels of IL-4 and IL-13 were quantified using high sensitivity ELISA kits from Invitrogen and Activin A and IL-2 using ELISA kits from Sigma Aldrich.

### Statistical Analysis

Statistical analyses were performed using Prism GraphPad Software version 8.0.2 and the statistical software R (20). After confirming a non-Gaussian distribution within the majority of the parameters analyzed, the Wilcoxon-Mann–Whitney test was used to compare continuous factors between two groups. Correlation analyses were performed using the Spearman rank correlation method with exact permutation *p*-values calculated. All *p*-values are two-sided, and a *p*-value of <0.05 was considered significant. In scatterplots, median values are indicated. To analyse the effect of the plasma markers measured (IL-4, IL-13, IL-5, CXCL13, Activin A, IL-2) on neutralization breath, we use a generalized linear model assuming a Poisson distribution (21, 22).

## Results

### Plasma CXCL13 Correlates With Disease Progression but Not Neutralization Breadth in HIV-Infected Children

**Figure 1:**
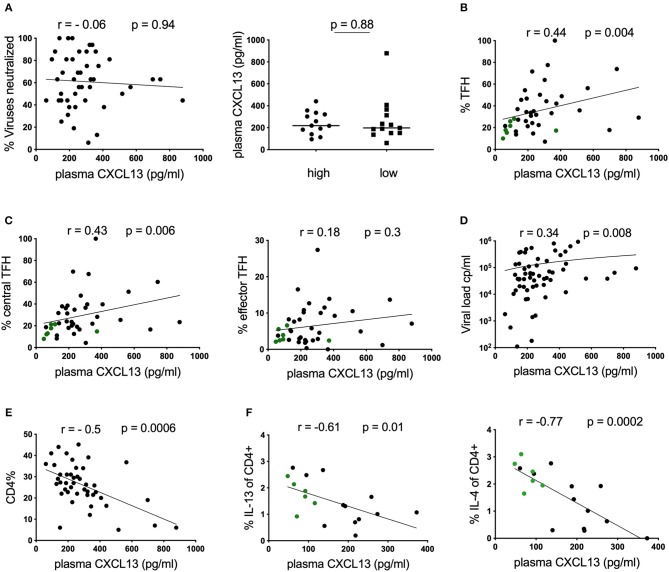
Plasma CXCL13 does not correlate with neutralization breadth in HIV-infected children. **(A)** Left: No correlation between plasma CXCL13 and neutralization breadth at matching time points in a cohort of HIV-infected children (*n* = 45). Right: No differences in plasma CXCL13 levels between children with high neutralization breadth (neutralization of ≥81% of viruses tested or ≥75% percentile; *n* = 13) and children with low neutralization breadth (neutralization of ≤44% of viruses tested or ≤25% percentile; *n* = 13). **(B)** Positive correlation between plasma CXCL13 and frequencies of circulating T-follicular helper cells (T_FH;_ CD4^+^CD45RA^−^CXCR5^+^CXCR3^−^PD1^+^) in children including HIV-ve controls with available samples (infected: *n* = 33: uninfected: *n* = 7, green dots) (*p* = 0.004, *r* = 0.44) (Table 1). **(C)** Circulating T_FH_ cells were further subdivided into circulating central T_FH_ cells (CCR7^+^; left) and circulating effector T_FH_ cells (CCR7^−^; right). Central T_FH_ cells but not effector T_FH_ cells were found to correlate significantly with CXCL13. **(D)** Positive correlation between CXCL13 and viral load and **(E)** an inverse correlation with CD4 percentage was observed in HIV-infected children (*n* = 45). **(F)** Inverse correlations between IL-13 and IL-4 production by bulk CD4 T cells using intracellular cytokine staining assays in response to PMA/Ionomycin stimulation in a subgroup of children including HIV-ve controls (infected: *n* = 12; uninfected *n* = 6, green dots) with available data. For comparison between 2 groups, Mann-Whitney tests were performed. Medians are indicated in scatter plots as a solid black line. Calculation of correlations were made by Spearman's rank correlation test.

To characterize potential plasma correlates of neutralization breadth, we first measured plasma CXCL13, a proposed marker of GC activity (9), in a cohort of antiretroviral (ART) naïve, HIV-infected children (*n* = 45) with available neutralization data from matching time points (4) (Table 1). In contrast to previous reports in adults (9), plasma CXCL13 were not correlated with HIV neutralization breadth when considering all individuals; or when comparing individuals in the top quartile of neutralizers (who neutralized ≥81% of tested viruses; “high neutralizers”) to those in the bottom quartile (who neutralized ≤44% neutralization breadth; “low neutralizers”; Figure 1A and Table 1). However, we did observe a correlation between the frequency of total blood T_FH_ cells (defined as CD3^+^CD4^+^CD45RA^−^CXCR5^+^CXCR3^−^PD1^+^ lymphocytes) and CXCL13 plasma levels (*r* = 0.44, *p* = 0.004; Figure 1B) within the whole pediatric cohort, including HIV-ve controls, suggesting a potential association between these parameters. Previously we observed that circulating effector T_FH_ cells (CCR7^−^) were associated with neutralization breadth, but central T_FH_ cells (CCR7^+^) were not (6). Examining these groups we find a correlation between CXCL13 and central T_FH_ cells (*r* = 0.43, *p* = 0.006), but not the functional effector T_FH_ subset (Figure 1C), consistent with lack of association between CXCL13 and neutralization breadth. Moreover, when excluding HIV uninfected children we find no significant associations between CXCL13 and any T_FH_ subset. We next examined markers of disease progression and found that plasma CXCL13 was directly correlated with plasma viral load (*r* = 0.34; *p* = 0.008; Figure 1D) and inversely correlated with CD4 percentage (*r* = −0.5; *p* = 0.0006; Figure 1E) and CD4 count (data not shown). Together these data suggest that, in HIV-infected children, CXCL13 plasma levels do not correlate with GC activity or T_FH_ functionality, but rather with HIV disease progression.

To further investigate this association, we assessed CD4 T-cell functionality in a subset of our cohort, for whom samples were available, by stimulating PBMCs with PMA/Ionomycin and measuring cytokine production in CD4 T-cells by intracellular cytokine staining. We observe a strong inverse correlation between the frequencies of cells producing the Th2 cytokines IL-4 and IL-13 and plasma CXCL13 (Figure 1F). Similar trends were also detected for TNF-α and INF-γ (Supplementary Figure S1), and maintained when only considering HIV-infected children (data not shown). Overall these data support the hypothesis that, in HIV-infected children CXCL13 is not a good marker of GC activity, but is rather associated with disease progression and loss of immune function.

### Plasma IL-5 Correlates With Neutralization Breadth in HIV-Infected Children

**Figure 2:**
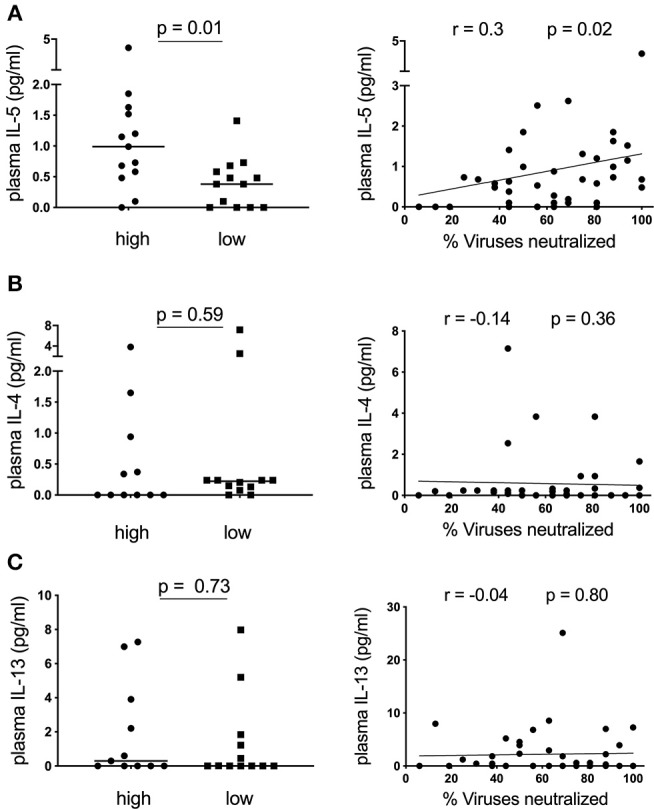
Plasma IL-5 correlates with neutralization breadth in HIV-infected children. **(A)** Left: Children with high neutralization breadth (neutralization of ≥81% of viruses tested or ≥75% percentile; *n* = 13) have significantly elevated levels of *ex-vivo* plasma IL-5 compared to children with low neutralization breadth (neutralization of ≤44% of viruses tested or ≤25% percentile; *n* = 13) (*p* = 0.01) (Table 1). Right: A positive correlation between plasma IL-5 and neutralization breath within HIV-infected children (*n* = 45) (*r* = 0.3, *p* = 0.02). **(B)** As of **(A)** but showing *ex vivo* plasma IL-4 (*n* = 43) and **(C)** IL-13 (*n* = 43) levels. Mann-Whitney test was performed. Medians are indicated in scatter plots as a solid black line. For correlations, calculations were made by Spearman's rank correlation test.

Having observed an association between plasma CXCL13 and the frequency of IL-4/IL-13 secreting CD4 T-cells, we next measured *ex-vivo* plasma levels of these cytokines and another canonical Th2 cytokine, IL-5, and determined their association with neutralization breadth. Surprisingly, of these cytokines only IL-5, (Figure 2A) but not IL-4 and IL-13 (Figures 2B,C), was elevated in children with high neutralization breadth (≥75% percentile, neutralization of ≥81% of viruses tested, *n* = 13) compared to those with low neutralization breadth (≤25% percentile, neutralization of ≤44% of viruses tested, *n* = 13) (*p* = 0.01). This corresponded with a positive correlation between plasma IL-5 and neutralization breadth in HIV-infected children (*r* = 0.3, *p* = 0.02). To account for multiple statistical comparisons, we conducted a multivariate Poisson regression model analysis for all cytokines measured, and confirmed a significant positive association between plasma IL-5 and neutralization breadth (*p* = 0.0038). There was, however, no correlation between plasma IL-5 and any of the circulating T_FH_ subsets or plasma CXCL13 (Supplementary Figure S2A) and overall little IL-5 production of CD4 T-cells when stimulated with PMA/ Ionomycin in a subset of the cohort with available samples (Supplementary Figure S2B). In conclusion, plasma IL-5, but not IL-4 or IL-13, correlates with neutralization breadth in HIV-infected children.

### No Association Between Plasma Activin A and Neutralization Breadth in HIV-Infected Children

**Figure 3:**
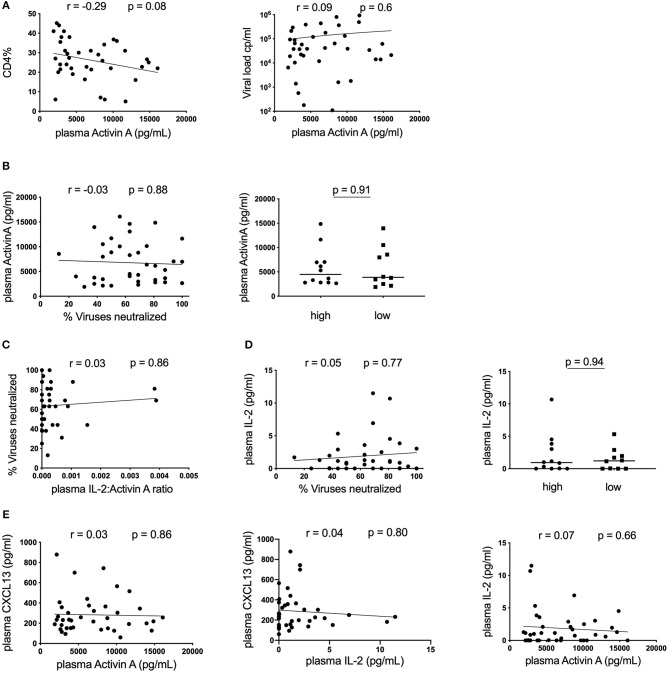
No association between plasma Activin A and neutralization breadth in HIV-infected children. **(A)** Left: Correlation between CD4% and plasma Activin A and Right: Lack of correlation between plasma Activin A and viral load in HIV-infected children (*n* = 39). **(B)** Left: No correlation between plasma Activin A and neutralization breadth in the same cohort. Right: No differences in plasma Activin A levels between children with high neutralization breadth (neutralization of ≥81% of viruses tested or ≥75% percentile; *n* = 13) and children with low neutralization breadth (neutralization of ≤44% of viruses tested or ≤25% percentile; *n* = 13). **(C)** Lack of correlation between neutralization breadth and the ratio of plasma IL-2:Activin A (*n* = 39). **(D)** Left: No correlation between IL-2 levels and neutralization breadth (*n* = 39). Right: No differences in plasma IL-2 levels between children with high (*n* = 13) and low neutralization breadth (*n* = 13). **(E)** Right: No correlation between plasma Activin A and plasma CXCL13. Middle: No correlation between IL-2 and CXCL13 in plasma. Right: No correlation between plasma IL-2 and plasma Activin A levels (*n* = 39). For comparison between 2 groups, Mann-Whitney tests were performed. Medians are indicated in scatter plots as a solid black line. For all correlations, calculations were made by Spearman's rank correlation test.

Finally, we assessed a novel cytokine associated with T_FH_ activity, Activin A. This molecule has been demonstrated to be a potent activator of T_FH_ differentiation through a mechanism that is antagonized by IL-2 (17). We therefore measured plasma levels of both Activin A and IL-2 in our cohort of HIV-infected children. To our knowledge, Activin A levels have not been assessed in HIV-infected individuals and the impact of disease on this cytokine was unknown. All individuals had high levels of Activin A in their plasma, ranging between 2133 and 16,088 pg/ml. Production of this cytokine may be altered by HIV infection as plasma levels correlated weakly with CD4% and a similar trend was observed for CD4 count (data not shown). However, this did not reach significance and no correlation with VL was observed (Figure 3A). Importantly, although there is a broad range of Activin A expression, we observed no correlation with neutralization breath in the cohort as a whole, and no significant difference in Activin A levels between the top 25% (high) and the bottom 25% (low) of subjects based on neutralization breadth (Figure 3B). Because effect of Activin A on T_FH_ is reported to be antagonized by IL-2, we also tested the association between the ratio of IL2:Activin A and neutralization breath and found no correlation (Figure 3C). Moreover, plasma IL-2 alone does not correlate with neutralization breadth (Figure 3D). Finally, there was no correlation between either Activin A or IL-2 and plasma levels of CXCL13 suggesting these cytokines are not co-regulated (Figure 3E).

## Discussion

The immune responses of children offer some unique perspectives that have the potential to inform HIV vaccine efforts (1). A protective HIV vaccine will most likely require the generation of bnAbs and this depends on an both an effective GC response and T_FH_ activity. Since direct monitoring of the GC response in future pediatric vaccine trials is not feasible due to lack of routine access to tissue, a plasma marker of the GC response, T_FH_ activity, and neutralization breadth would be of great value. While in adults plasma CXCL13 levels during early HIV infection can predict the generation of bnAbs in chronic disease stage (8, 10) and individuals with high neutralization breadth show elevated CXCL13 levels (9), this had not previously been examined in pediatric patients.

Our observations that plasma CXCL13 correlated with disease progression and general immune activation in pediatric patients rather than with neutralization breadth is in line with previously published findings where plasma CXCL13 was elevated in adults with progressive HIV infection and correlated with the inflammation-associated chemokine IP-10 (23). Furthermore, plasma CXCL13 levels were elevated in HIV-infected children compared to HIV-ve controls and correlated with viral load in infected children (16, 24). In adults, bnAb development is linked with viral loads and therefore with disease progression (25), whereas in pediatrics bnAb development is also facilitated by functional T-follicular helper and regulatory responses in the setting of persistent high viremia (4, 6). Additionally, plasma CXCL13 has been associated with immune activity in various infectious and autoimmune diseases (23, 26–30), suggesting that it may not be a specific marker of germinal center activity. This is supported by our observation that plasma CXCL13 correlated with total circulating T_FH_ but not the effector T_FH_ subset previously shown to be associated with neutralization breadth in HIV-infected children (6). A potential caveat here is that our correlations between circulating T_FH_ and plasma CXCL13 levels include HIV-ve controls, hence not limiting our observations to the dynamics in HIV infection. Interestingly, in adult viremic controlers, an inflammatory cytokine signature including CXCL13 and IP-10 was associated with the development of bnAbs (31). These data again demonstrate that interplay between virus, immune activation and neutralization breadth in pediatric patients appears to be fundamentally distinct from adult infection (4). It is important to note, however, that CXCL13 may still have value as a marker of GC activity in the setting of childhood vaccination, which was not tested here.

The finding that plasma IL-5, but not the other classic Th2 cytokines IL-13 and IL-4, correlated with neutralization breadth within our cohort of HIV-infected children was unexpected. Impaired IL-5 production of T-cells has been previously described as a prognostic markers of disease progression in HIV-infected children (19). In this present study, we did not observe significant differences between the different clinical groups and IL-5 production of CD4 T-cells was very limited in general. It is therefore probable that other cell subsets are important sources of plasma IL-5, including type 2 innate lymphoid cells, mast cells, eosinophils and/or neutrophils (32, 33). Future work will be required to identify the source of IL-5 and may shed light on immune pathways that support antibody maturation. However, recently published data support an association between circulating CD8^+^CD57^+^ T-cells in viremic controllers and neutralization breadth (34) and these cells were previously found to secrete high levels of IL-5 (35). In addition, IL-5 is known to be essential for antibody class switching in mice (36) and stimulates B-cell proliferation (37), particularly of germinal center B-cells (18). Finally, the fact that plasma levels of IL-5 correlated with neutralization breadth in HIV-infected children may also point to increased regulatory activity in these individuals. IL-5 promotes the expansion and survival of antigen specific T regulatory cells (Treg) (38), and treatment with recombinant IL-5 leads to an expansion of Treg and reduces pathology in experimental autoimmunity (39). Indeed, the fact that we observe no correlation between plasma IL-5 and circulating effector T_FH_, might suggest that the role for IL-5 does not relate to the germinal center activity. However, further work in an extended cohort of individuals or in the animal model is required to test these hypotheses. It would also be interesting to evaluate additional predictive plasma markers of neutralization breadth as has been done for adult infection (8, 10).

A biomarker which predicts GC activity, T_FH_ activity, and neutralization breadth in the pediatric population would offer a powerful tool for vaccine development. Our results suggest that IL-5 is a potential candidate. In addition, exploration of the biology behind this association may improve our understanding of how broadly neutralizing antibodies develop in this key population.

## Data Availability

All relevant datasets generated for this study are included in the manuscript and/or the Supplementary Files.

## Ethics Statement

Informed consent was obtained from all adult study participants or from the caregivers of pediatric participants where appropriate. Additionally, assent to participate in the study was given directly by children from the age of 6 and above. Studies were approved by the University of the Free State Ethics Committee, Bloemfontein; Biomedical Research Ethics Committee, University of KwaZulu-Natal, Durban; and Research Ethics Committee, University of Oxford.

## Author Contributions

JR designed the study, conducted experimental work within the study, analyzed the data, and wrote the paper. JP conducted experimental work within the study and analyzed the data. PO conducted experimental work within the study. MM and EA recruited subjects and supervised the study cohort. AG performed statistical analyses. TN, LM, and PM supervised experimental work within the study. HK, PG, and AL supervised experimental work within the study, analyzed data, and wrote the paper. PG established research cohorts.

### Conflict of Interest Statement

The authors declare that the research was conducted in the absence of any commercial or financial relationships that could be construed as a potential conflict of interest.
