# Supplementary material for: Plasma IL-5 but Not CXCL13 Correlates With Neutralization Breadth in HIV-Infected Children
Source: Front Immunol. 2019 Jul 2;10:1497. doi: 10.3389/fimmu.2019.01497 (PMC6615198; doi:10.3389/fimmu.2019.01497)
Supplement: Supplementary file 1 [file Table_1.DOCX]

Supplementary Material

# Supplementary Figures

**Fig S1**

**S1 Fig. Plasma CXCL13 correlates inversely with cytokine production of CD4 T-cells.** Inverse correlations between INF-γ (left) and TNF-α (right) production of bulk CD4 T cells in a subgroup of children independent of HIV infection (infected: n=12; uninfected n=6; green dots) with available data from intracellular cytokine staining assays in response to PMA/ Ionomycin stimulation. Statistical calculations were made by Spearman’s rank correlation test.

**Fig S2**

**S2 Fig. Lack of correlation between plasma IL-5 and circulating T_FH_ subsets. (A)** Lack of correlation between plasma IL-5 and circulating T_FH_ subsets as well as plasma CXCL13 levels in children including HIV-ve controls with available samples (infected: n=44; uninfected: n=5 , green dots) (Table 1). **(B)** IL-5 production by bulk CD4 T cells using intracellular cytokine staining assays in response to PMA/Ionomycin stimulation in a subgroup of children with available samples (children with high neutralization breadth; n=6 versus children with low neutralization breadth; n=4).
